# In Vitro Antioxidant Activities of Phenols and Oleanolic Acid from Mango Peel and Their Cytotoxic Effect on A549 Cell Line

**DOI:** 10.3390/molecules23061395

**Published:** 2018-06-08

**Authors:** Xuelian Bai, Tongfei Lai, Ting Zhou, Yicong Li, Xiuting Li, Huawei Zhang

**Affiliations:** 1 College of Life and Environmental Sciences, Hangzhou Normal University, Hangzhou 310036, China; baixl2012@163.com (X.B.); laitongfei@163.com (T.L.); tingzhou@163.com (T.Z.); yicong@126.com (Y.L.); 2 Beijing Advanced Innovation Center for Food Nutrition and Human Health, Beijing Technology and Business University (BTBU), Beijing 10048, China; 3 School of Pharmaceutical Sciences, Zhejiang University of Technology, Hangzhou 310014, China

**Keywords:** mango peel, phenol, antioxidant effect, cytotoxic activity

## Abstract

Mango peel, the main by-product of juice processing, possesses appreciable quantities of bioactive phenolic compounds and is worthy of further utilization. The present work reports for the first time the HPLC analysis and in vitro antioxidant evaluation of mango peel phenols (MPPs) and their cytotoxic effect on the A549 lung cancer cell line. These results indicated that mango peel has the total phenolic content of 723.2 ± 0.93 mg·kg^−1^ dry mango peel (DMP), which consisted mainly of vanillic aldehyde, caffeic acid, chlorogenic acid, gallic acid, procyanidin B_2_ and oleanolic acid. Antioxidant assays showed that MPPs had strong antioxidant activities, with 92 ± 4.2% of DPPH radical scavenging rate, 79 ± 2.5% of ABTS radical inhibition rate and 4.7 ± 0.5 μM Trolox equivalents per kg^−1^ DMP of ferric reducing power. Gallic acid possess a stronger antioxidant capacity than other phenols. In vitro cytotoxic tests suggested that mango peel extract (MPE) had an IC_50_ value of 15 mg·mL^−1^ and MPPs had a stronger inhibitory effect on the A549 cell line. Oleanolic acid exhibited the strongest cytotoxicity, with an IC_50_ value of 4.7 μM, which was similar with that of the positive control 5-fluorouracil.

## 1. Introduction

Mango is one of the most popular fruits, with high nutrition and commodity value, which is grown in over 70 countries in the world and includes about 35 species, including *Mangifera altissima*, *M. applanata*, *M. indica*, etc., which *M. indica* L. is one of the most cultivated varieties. As of 2005, China has been listed as the second largest producer of mango, with 12.9% of the world's output. Mango fruit is suitable for not only fresh eating, but also processing into juices, chutneys, sauces and preserved fruit. Worldwide this accounts for about 25% of mango production. However, the large amount of by-products, like mango skins mango seeds (representing 20 to 40% of the total mass), causes environmental pollution [1]. Recently, some researchers discovered that mango (*M. indica*) peel is a rich source of phenolic compounds, with amounts up to 0.55~1.1 g·kg^−1^ dry mango peel (DMP) [2,3]. Although many studies had been carried out on extraction and purification of mango phenols [4,5,6,7,8,9], few have reported on the compositional analysis, antioxidant and cytotoxic effects of mango peel phenols (MPPs). The present work is the first to describe an evaluation of the in vitro antioxidant activities of mango (*M. indica* L.) peel extract (MPE) and its MPPs, including DPPH•, ABTS•+ and FRAP scavenging, as well as their cytotoxic effect on the A549 human lung carcinoma cell line.

## 2. Results and Discussion

### 2.1. Total Phenolic Content and HPLC Analysis

**Figure 1:**
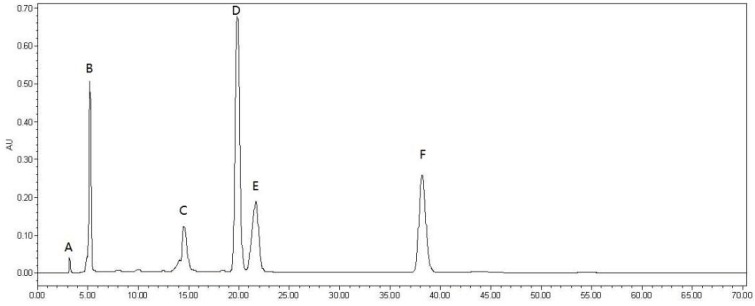
HPLC chromatography profile of mango peel extract (MPE). (**A**–vanillic aldehyde; **B**–caffeic acid; **C**–oleanolic acid; **D**–chlorogenic acid; **E**–gallic acid; **F**–procyanidin B_2_).

**Figure 2:**
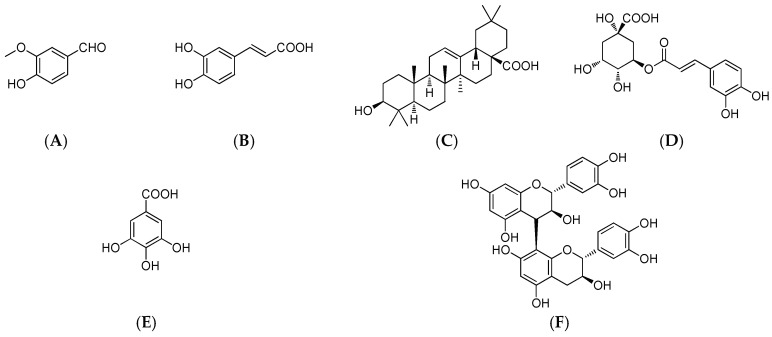
Chemical structures of detected compounds in MPE. (**A**–vanillic aldehyde; **B**–caffeic acid; **C**–oleanolic acid; **D**–chlorogenic acid; **E**–gallic acid; **F**–procyanidin B_2_).

Using the modified Folin–Ciocalteu method, the total phenolic content was determined to be 723.2 ± 0.93 mg·kg^−1^ DMP in MPE of *M. indica* L. As shown in Figure 1, five phenolic compounds were detected in MPE, including vanillic aldehyde (**A**), caffeic acid (**B**), chlorogenic acid (**D**), gallic acid (**E**), procyanidin B_2_ (**F**) together with oleanolic acid (**C**) (Figure 2). Quantitative analysis showed that mango peel had the highest content of chlorogenic acid (19.8 ± 0.31 mg·kg^−1^) followed by procyanidin B_2_ (12.5 ± 0.61 mg·kg^−1^), caffeic acid (11.4 ± 0.08 mg·kg^−1^), gallic acid (7.0 ± 0.57 mg·kg^−1^), oleanolic acid (3.0 ± 0.25 mg·kg^−1^), vanillic aldehyde (0.55 ± 0.04 mg·kg^−1^).

### 2.2. Antioxidant Activity of MPE and MPPs

An in vitro antioxidant assay indicated that MPE had strong capacity of scavenging DPPH and ABTS radicals and ferric reducing power at 2.0 mg·mL^–1^, which were 92 ± 4.2%, 79 ± 2.5%, 4.7 ± 0.53 μmol TE·kg^–1^ DMP, respectively. In order to identify the potential contributor(s) in MPE, six detected phenolic compounds were further evaluated their antioxidant effects. As shown in Table 1, these MPPs and oleanolic acid exhibited different antioxidant capacity at 100 µM. Especially, gallic acid possessed the strongest DPPH• scavenging rate (97 ± 0.4%), ABTS•+ inhibition rate (86 ± 3.0%) and reducing power (12 ± 0.5 µmol TE·kg^–1^ DMP), which was consistent with the findings revealed by Yen and his coworkers [10]. The antioxidant activity of oleanolic acid was weaker than that of any of the other chemical constituents in MPE, with 2.7 ± 0.17% of DPPH• scavenging rate, 11 ± 1.5% of ABTS•+ inhibition rate and 1.2 ± 0.09 µmol TE·kg^−1^ DMP of reducing power.

### 2.3. Cytotoxic Effect of MPE and MPPs

As shown in Table 2, MPE exhibited in vitro weak cytotoxic activity against A549 lung cancer cells, with an IC_50_ value of 15 mg·mL^−1^, while caffeic acid, oleanolic acid, chlorogenic acid and procyanidin B_2_ had potent inhibitory effects. Among these MPPs, oleanolic acid had the strongest cytotoxic effect, with an IC_50_ value of 4.7 μM, which was similar to that of the positive control 5-fluorouracil (3.8 μM).

**Figure 3:**
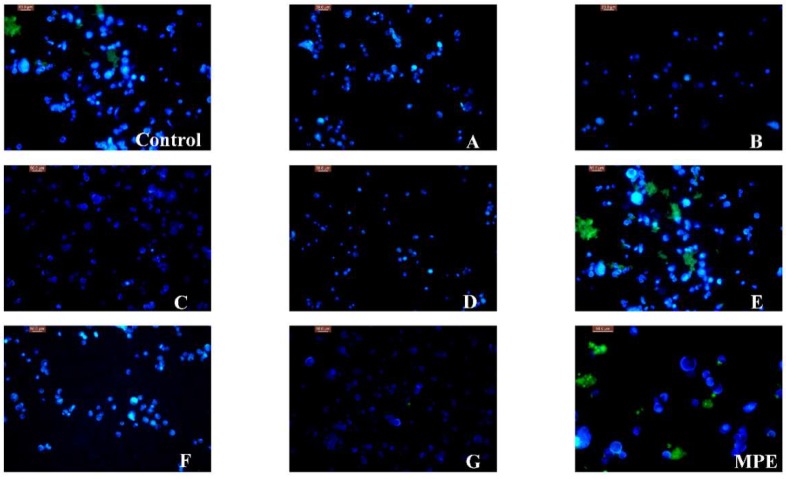
Fluorescence microscopy of A549 cell line treated with MPE, MPPs and oleanolic acid. (Control–untreated, **A**–vanillic aldehyde, **B**–caffeic acid, **C**–oleanolic acid, **D**–chlorogenic acid, **E**–gallic acid, **F**–procyanidin B_2_, **G**–5-fluorouracil, MPE–mango peel extract).

These findings were consistent with the morphological variations of the nucleus of A549 cells observed using 4′,6-diamidino-2-phenylindole (DAPI) staining. As shown in Figure 3, the nucleus showed weak blue fluorescence when A549 cells were treated with caffeic acid, oleanolic acid and chlorogenic acid, which suggested that these chemicals had significant in vitro cytotoxic effects. However, the strong fluorescence of the nucleus treated with MPE and gallic acid indicated they had weak antiproliferative activity.

## 3. Experimental Section

### 3.1. Determination of Total Phenolic Content

Fresh mango (*M. indica* L.) was purchased from a fruit market in Hangzhou (China). After peeling, lyophilizing and grinding, about 20 g of DMP powder was extracted with 200 mL of 70% aqueous ethanol (50 °C) under stirring for 20 min using microwave-assisted extraction (MAS-I, Xinyi Microwave Equipment Co., Ltd, Shanghai, China). The obtained MPE was concentrated under reduced pressure and lyophilized (LGJ-10N, Beijing Yaxing Instrument Co., Ltd., Beijing, China). After redissolving in 500 mL of 20% ethanol solution, the resulting extract was centrifuged for 15 min at 60,000× *g* (CR21GII, Hitachi Koki Co., Ltd., Tsuen Wan, Japan) and retained on a normal atmosphere column with 200 mL macroporous absorbent resin XAD-16 (Rohm and Haas, Philadelphia, PA, USA) followed by eluting with three bed volumes of 80% alcohol in H_2_O. The collected elution was concentrated under reduced pressure and lyophilised. The afforded phenol-enriched extract was dissolved in methanol with a final concentration of 20 mg·mL^−1^ and preserved at 4 °C before HPLC analysis. The total phenolic content of MPE was determined by the modified Folin–Ciocalteu method proposed by Wijngaard and Brunton [11]. Triplicate tests were conducted for each sample. The result was expressed as gram of gallic acid equivalents per kilogram of DMP (g GAE·kg^−1^ DMP).

### 3.2. HPLC Analysis of MPPs and Oleanolic Acid

Five phenolic compounds, vanillic aldehyde, caffeic acid, chlorogenic acid, gallicacid, procyanidin B_2_, and oleanolic acid (Figure 2), and 5-fluorouracil were purchased from Sigma-Aldrich Chemical (St. Louis, MO, USA) and respectively dissolved in methanol to a final concentration of 100 μM. HPLC analysis of MPPs was performed using a HPLC system (Shimadzu, Toyko, Japan) equipped with a SIL-10AF autosampler, a CTO-10AS column oven (25 °C) and a SPD-10AV UV-visible detector on a reversed-phase SunFire C8 (250 mm × 4.6 mm, ID 5 µm) column (Waters, Milford, MA) under an isocratic condition of 10% acetonitrile in H_2_O with 0.1% acetic acid at 1.0 mL·min^−1^ and 280 nm. All solvents were filtered with a 0.22 µm membrane filter and each injection volume was 10 µL. Quantitative analysis of MPPs and oleanolic acid was carried out using the external standard method described before [12]. Triplicate tests were conducted for each sample. The level of each compound was expressed in mg·kg^−1^ DMP.

### 3.3. Antioxidant Assay of MPPs

#### 3.3.1. DPPH Assay

MPP (100 µL) or oleanolic acid (100 µM) or MPE (2.0 mg·mL^−1^) solution were mixed with 2 mL of 2,2-diphenyl-1-picrylhydrazyl radicals (DPPH, Sigma-Aldrich, 0.1 mM in methanol). The reaction mixture was shaken vigorously and stored in the dark for 30 min at room temperature. The absorbance of the solution was then recorded on a Hitachi-UV-3000 spectrophotometer (Hitachi, Tokyo, Japan) at 517 nm. 100 µL of methanol was added to the DPPH solution used as a blank control. All measurements were performed in triplicate. The percentage scavenging effect was calculated as follows:

$$I_{DPPH}=\frac{A_{0}-A_{1}}{A_{0}}\times 100\%$$

where *I_DPPH_* is the DPPH radical scavenging rate (%), *A*_0_ is the absorbance of a negative control, *A*_1_ is the absorbance of each sample [13].

#### 3.3.2. ABTS Assay

The capability of scavenging 2,2′-azinobis(3-ethylbenzothiaziline-6-sulfonate radical (ABTS, Sigma-Aldrich) radical was carried out according to the method described by Bao and his co-workers [14]. The ABTS radical was prepared by mixing equal volumes of 7 mM aqueous ABTS and 2.45 mM aqueous potassium persulfate. The solution was kept in the dark at room temperature for 16 h before use. The absorbance of ABTS radical solution at 734 nm was adjusted to 0.7 by dilution with distilled water. 1 mL of fresh ABTS•+ solution was added to 10 mL sample solution or methanol (negative control), and stored at room temperature for 6 min. The absorbance of the reaction mixed solution was immediately measured at 734 nm. All measurements were run in triplicate. The percentage decrease of the absorbance at 734 nm was calculated by the formula:

$$I_{ABTS}=\left[\frac{A_{0}-A_{1}}{A_{0}}\right]\times 100\%$$

where *I_ABTS_* is the ABTS•+ inhibition rate (%), *A*_0_ is the absorbance of a negative control, *A*_1_ is the absorbance of each chemical (100 µM) or MPE (2.0 mg·mL^-1^) solution.

#### 3.3.3. FRAP Assay

Fresh FRAP reagent was prepared by mixing 2.5 mL of tripyridine triazine (TPTZ, Sigma-Aldrich, 10 mM in 40 mM HCl), 2.5 mL of ferric chloride (20 mM) and 25 mL of sodium acetate buffer (300 mM, pH 3.6). 900 µL of the FRAP reagent were mixed with 300 µL of each phenol (100 µM) or MPE (2.0 mg·mL^−1^) solution or methanol (negative control) in 1 cm disposable plastic cells and incubated at 37 °C for 120 min. The absorbance was taken on a Hitachi-UV-3000 spectrophotometer at 595 nm. A standard curve was obtained using Trolox solution at 0.5, 1.0, 1.5, 2.0 mM. The absorbance of each sample was compared with that of the Trolox standard and the results were expressed in terms of micromole Trolox equivalents per kg DMP (µM TE·kg^−1^ DMP). All measurements were performed in triplicate. The ferric reducing power was calculated by the following equation:

$$I_{FRAP}=\frac{A_{1}}{A_{0}}\times 100\%$$

where *I_FRAP_* is the ferric reducing power, *A*_0_ is the absorbance of a negative control, *A*_1_ is the absorbance in the presence of a tested sample [15].

### 3.4. Cytotoxic Effect of MPPs and Oleanolic Acid on the A549 Cell Line 

#### 3.4.1. Inhibitory Activity

Human NSCLC cell lines A549 were obtained from the Cancer Institute of Sun Yat-Sen University (Guangzhou, China) and cultured in RPMI-1640 (Sangon Biotech Co., Ltd., Shanghai, China) medium containing 10% FBS (Sangon Biotech Co., Ltd.) and 1% (0.01 g·mL^−1^) penicillin-streptomycin (Sigma-Aldrich, St. Louis, MO, USA) at 37 °C in a humidified 5% CO_2_ incubator (Chengdu Must Bio-Technology Co., Ltd., Chengdu, China). The medium was changed daily and subcultured when they reached a confluence of 80%.

Six chemicals including vanillic aldehyde, caffeic acid, oleanolic acid, chlorogenic acid, gallic acid and anthocyanidin B_2_ were dissolved in 500 μL DMSO solution, filtered by the filter membrane of 0.22 μm. These solutions were diluted with liquid containing 10% fetal bovine serum RPMI-1640 (Sangon Biotech Co., Ltd.) culture resulting in final concentrations of 6.25, 12.5, 25, 50, 75, 100, 150, 200 μM, respectively. MPPs and oleanolic acid were also dissolved in accordance with the above methods in final concentrations of 68.5, 100, 200, 300, 400, and 500 μg·mL^−1^, with 5-fluorouracil as the positive control.

The effect of MPPs and oleanolic acid on cell proliferation of A549 was evaluated by cell counting kit-8 (CCK-8) assay according to the manufacturer’s instruction. Briefly, cells in 96-well plates at a density of 5 × 10^3^ per well were incubated for 24 h at 37 °C. Then the supernatant was removed and the cells were treated for 24 h with chemicals at different concentrations (6.25, 12.5, 25, 50, 75, 100, 150, 200 μM) or MPPs at different concentrations (68.5, 100, 200,300, 400, and 500 μg·mL^−1^). CCK-8 (10 μL) dissolved in PBS (5 mg·mL^−1^) was added to each well, and the cells were further incubated for 45 min. Finally, the medium containing CCK-8 was removed and 100 μL of DMSO was added to each well. The plate was gently shaken for 10 min to dissolve the formazan crystals (Sangon Biotech Co., Ltd.) and the absorbance was measured at 450 nm on a ELISA (Thermo Electron Co., Vantaa, Finland). The percentage viability was calculated using the following formula:

$$IC=\left[1-\frac{{OD}_{sample}-{OD}_{blank}}{{OD}_{negative}-{OD}_{blank}}\right]\times 100\%$$

Sample group (90 µL cell suspension + 10 µL phenols solution + 10 µL CCK-8 reagent (Sangon Biotech Co., Ltd.), blank group (90 µL cell medium + 10 µL sample solution + 10 µL CCK-8 reagent), negative groups (90 µL cell suspension + 10 µL cell medium + 10 µL CCK-8 reagent). The concentration of MPPs and oleanolic acid needed to inhibit cell growth by 50% (IC_50_) was calculated from the dose response curves for each cell line [16].

#### 3.4.2. Morphological Observation of the Nucleus of Lung Cancer A549 Cells

The logarithmic phase of A549 cells, using 0.25% of trypsin digestion, counting, add broth, made 5×10^4^/mL cells suspension, vaccination in culture bottle, set up the experimental group and positive control group and blank group, respectively. Each sample was incubated at 37 °C for 24 h to join testing solution (100 μM) 1 mL and MPPs and oleanolic acid (100 μg·mL^−1^), 5-fluorouracil (100 μM, Sigma-Aldrich) as the positive control group and blank group, added 1 mL cultures and incubated at 37 °C for 48 h [17,18]. The experimental group, positive control group and blank group of medium, with sterile PBS washing, with trypsin digestion cells, using RPMI 1640 culture medium containing 10% fetal bovine serum termination of digestion, made the cell suspension, the cell suspension put in centrifuge tube, 1000 r/min, the centrifugal 5 min, abandon supernatant. Plus 5 μL DAPI (0.2 mg·mL^−1^) (Sigma-Aldrich) and kept in dark at 37 °C for 30 min, after the incubation, washing with buffer, add 1 mL culture, in the inverted fluorescence microscope observed under fluorescent green fluorescence set (Olympus Co., Tokyo, Japan) and take photos, and use the Image-ProPlus 5.1 software (Media Cybernetics, Rockville, MD, USA) processing images, green fluorescence intensity ratio calculation [19,20].

### 3.5. Statistical Analysis

All data were expressed as mean ± standard deviation (SD) and analyzed by SPSS software (Version 19.0, Chicago, CA, USA). Values of *p* < 0.05 were considered significant.

## 4. Conclusions

The present work firstly reported on in vitro antioxidant activities of phenols and oleanolic acid from mango peel and their cytotoxic effect on the A549 cell line. The ethanolic extract of mango peel is rich in phenolic compounds. HPLC analysis indicated that MPE had five main chemical constituents, including vanillic aldehyde, caffeic acid, chlorogenic acid, gallic acid, procyanidin B_2_ and oleanolic acid. Antioxidant assays suggested that MPE had strong DPPH and ABTS radical scavenging capacity and ferric reducing power at 2.0 mg·mL^−1^. Gallic acid possessed the strongest DPPH• scavenging rate (97 ± 0.4%), ABTS•+ inhibition rate (86 ± 3.0%) and reducing power (12 ± 0.5) µmol TE·kg^−1^ DMP. In vitro CCK-8 assay indicated that oleanolic acid exhibited the strongest inhibitory effect on A549 cell line with an IC_50_ value of (4.71 ± 0.30 μM). Fluorescence staining experiments confirmed that oleanolic acid inhibited cell proliferation by destroying the nucleus of A549 cell. These findings would promote the utilization of mango peel and the development of antioxidant food.

## Figures and Tables

**Table 1:** Antioxidant activity of six phenol compounds (*n* = 3).

Phenol	Antioxidant Activity		
	DPPH• Scavenging Rate (%)	ABTS• + Inhibition Rate (%)	FRAP Assay (TE, µmol·kg^−^^1^)
**A**	60 ± 1.0	80 ± 3.2	8.0 ± 0.96
**B**	29 ± 1.0	55 ± 4.5	6.0 ± 0.65
**C**	2.7 ± 0.17	11 ± 1.5	1.2 ± 0.09
**D**	80 ± 1.2	76 ± 3.6	8.0 ± 0.25
**E**	97 ± 0.4	86 ± 3.0	12 ± 0.52
**F**	78 ± 1.2	76 ± 2.4	8.0 ± 0.22
MPE *	92 ± 4.2	79 ± 2.5	4.7 ± 0.53

(**A**—vanillic aldehyde, **B**—caffeic acid, **C**—oleanolic acid, **D**—chlorogenic acid, **E**—gallic acid, **F**—procyanidin B_2_, **G**—5-fluorouracil, MPE—mango peel extract) * at 2.0 mg·mL^−1^.

**Table 2:** In vitro cytotoxic effect of MPE, MPPs and oleanolic acid on A549 cells.

	A	B	C	D	E	F	G	MPE
IC_50_ value (μM)	7.2	8.9	4.7	9.8	51.8	14.3	3.8	15 mg·mL^−^^1^

(**A**–vanillic aldehyde, **B**–caffeic acid, **C**–oleanolic acid, **D**–chlorogenic acid, **E**–gallic acid, **F**–procyanidin B_2_, **G**–5-fluorouracil, MPE–mango peel extract).
